# Assessment of PD-L1 mRNA and protein expression in non-small cell lung cancer, head and neck squamous cell carcinoma and urothelial carcinoma tissue specimens using RNAScope and immunohistochemistry

**DOI:** 10.1371/journal.pone.0215393

**Published:** 2019-04-15

**Authors:** David Jonathan Duncan, Marietta Scott, Paul Scorer, Craig Barker

**Affiliations:** Precision Medicine Laboratories, Precision Medicine and Genomics, Innovative Medicines and Early Development Biotech Unit, AstraZeneca, Cambridge, United Kingdom; University of South Alabama Mitchell Cancer Institute, UNITED STATES

## Abstract

Four immunohistochemistry (IHC) diagnostic assays have been approved for tumour PD-L1 protein assessment in the clinic. However, mRNA detection by in situ hybridisation (ISH) could be utilised as an alternative to protein detection. Detecting spatial changes in gene expression provides vital prognostic and diagnostic information, particularly in immune oncology where the phenotype, cellular infiltration and immune activity status may be associated with patient survival. Translation of mRNA expression to a clinically relevant cut off or threshold is challenging due to variability between assays and the detection of different analytes. These studies aim to confirm the suitability of formalin fixed paraffin embedded (FFPE) tissue sections for use with RNA ISH. A comparison of mRNA expression and protein expression may inform the suitability of mRNA as a patient selection biomarker in a similar manner to IHC and provide evidence of a suitable scoring algorithm. Ninety patient samples, thirty for each indication of non-small cell lung cancer (NSCLC), head and neck squamous cell carcinoma (HNSCC) and urothelial carcinoma (UC), previously assessed using the VENTANA PD-L1 (SP263) Assay were chosen to represent a wide dynamic range of percentage tumour cell staining (TC_IHC_). Expression of mRNA was assessed by ISH using the RNAScope 2.5 assay and probe CD274/PD-L1 (Advanced Cell Diagnostics) including kit provided positive and negative control probes. Brightfield whole slide images of tissues were captured. The percentage of tumour cells with PD-L1 mRNA expression (%TC_mRNA_) and mean punctate dots/tumour cell were determined using image analysis. Differences in RNA expression between the IHC derived TC_IHC_≥25% and <25% groups were assessed using t-tests. For each indication, a receiver-operating characteristic (ROC) analysis identified thresholds for patient classification using %TC_mRNA_ and dots/tumour cell, with reference to TC_IHC_≥25%. Eighty-six samples were successfully tested; 3 failed due to insufficient control probe staining, 1 due to lack of tumour. Percent TC_mRNA_ staining using RNAScope demonstrated statistical significance (at α = 0.05) in the PD-L1 high (TC_IHC_ ≥25%) vs the PD-L1 low (TC_IHC_ <25%) groups for NSCLC, HNSCC, and UC. The number of punctate dots/tumour cell was significantly higher in the PD-L1 high vs the PD-L1 low groups for NSCLC and HNSCC but not UC. For %TC_mRNA_; ROC analysis identified thresholds of: NSCLC 18.0%, HNSCC 31.8%, UC 25.8%. For dots/tumour cell, thresholds were: NSCLC 0.26, HNSCC 0.53, UC 0.45. Routine tissue fixation and processing is suitable for RNA detection using RNAScope. PD-L1 mRNA extent and level is associated with PD-L1 status determined by IHC. Threshold optimisation for %TC_mRNA_ and mean dots/tumour cell results in high specificity to IHC PD-L1 classification, but only moderate sensitivity.

## Introduction

Programmed cell death ligand 1 (PD-L1) is part of a complex system of receptors and ligands that are involved in controlling T cell activation. PD-L1 acts at multiple sites in the body to help regulate immune responses by delivering inhibitory signals to T cells through the programmed cell death 1 (PD-1) and cluster of differentiation (CD) 80 receptors. PD-L1 is a member of the B7 family of ligands that inhibit T-cell activity through binding to the PD-1 receptor [1] and to CD80 [2]. Expression of PD-L1 protein is under the control of inflammatory signals that are typically associated with an adaptive immune response (e.g. interferon gamma [IFNγ]) and can be found on both tumour cells and tumour infiltrating immune cells. The binding of the ligand PD-L1 to its receptor PD-1 on activated T cells delivers an inhibitory signal to the T cells, preventing them from killing target tumour cells, and protecting the tumour from immune elimination [3].

Numerous therapies have been approved or are undergoing investigation which target either PD-1 or PD-L1 and elicit anti-tumour activity [4]. The binding of an anti-PD-L1 therapeutic agent to the PD-L1 receptor inhibits the interaction of PD-L1 with the PD-1 and CD80 receptors expressed on immune cells; restoring the host immune response, a mechanism which has been extensively reviewed [5, 6]. The impact of PD-L1 and PD-1 expression on tumour cells and immune cells within the tumour microenvironment has been well studied and reviewed, PD-L1 has been shown to act on both tumour cells and immune cells preventing tumour cell lysis by T-cells [7]. The activity of PD-1/PD-L1 therapy overcomes PD-L1-mediated inhibition of antitumor immunity.

The patient population may be stratified by diagnostic assays to determine those patients most likely to respond to IO therapy. Additionally, diagnostic assays may also provide data to manage the expectation of tumour response, adverse effects and tolerability. These assays are typically immunohistochemistry (IHC) assays co developed during clinical trials and at the time of writing, the only regulatory approved companion diagnostic assays for PD-L1 detection are IHC based [8, 9]. In addition, assays which measure tumour mutational burden, microsatellite instability and the immunogenicity of tumours are under development to stratify patients for immuno-oncology treatment [10].

Multiple IHC PD-L1 assays have been developed for use with immunotherapies; each with different cut-offs and scoring algorithms. The ability to interchange assays facilitates PD-L1 testing for patient eligibility. Different assays show varying concordance, for example Ratcliffe et al [11] demonstrated good agreement between three diagnostic assays used in non-small cell lung cancer (NSCLC), according to manufacturer’s instructions. Strong intrinsic agreement has been shown in NSCLC and head and neck squamous cell carcinoma (HNSCC) using SP263, 22C3 and 28–8 IHC assays [12]. More recently, assessment of 11 laboratory developed tests to detect PD-L1 by IHC in NSCLC has shown 6 of the protocols demonstrated good concordance with regulatory approved commercial assays using 22C3 and 28–8 primary antibodies [13].

Assays which detect alternative molecules to protein have been investigated across pathologies and in different indications including those which measure mRNA or DNA by *in situ* hybridisation (ISH). ISH has been shown to be an alternative to IHC, for example for the detection of PD-L1 [14, 15] and for the detection of other drivers of tumorigenesis such as *HER2* amplification [16] PTEN [17] and *MET* amplification [18]. These assays have also been shown to detect spatial changes in gene expression which could provide vital prognostic or diagnostic information in different patient populations [15]. Detecting biomarkers *in situ* offers an insight into the heterogeneity of tissue. Morphology of tissue may impact RNA expression and could possibly vary in neighbouring cells. Quantifying the amount of gene expression may provide additional sensitivity and specificity over other assays, offering greater predictive power for patient outcome and/or stratification, potentially increasing the patient population that will respond to targeted therapy [16, 19].

RNAScope is a commercially available, fully automated, ISH assay for the detection of RNA in a variety of sample types including formalin fixed paraffin embedded tissue [20, 21]. The RNAScope assay visualises RNA by developing a chromogen to produce small punctate dots which are quantitative and therefore less analytically subjective. RNAScope has been used to detect PD-L1 RNA and has been compared to other assays such as IHC in various indications including small cell lung cancer (SCLC) [15]; positivity using RNAScope was defined in this study as the presence of 4–10 punctate dots per tumour cell. RNAScope positivity was compared to IHC positivity, defined as a tumour proportion score (percentage of cells with partial or complete cell membrane staining at any intensity) of greater than 1% using Dako 28–8 and Ventana SP142 antibodies. PD-L1 prevalence using RNAScope and IHC assays were similar (15.5% vs 16.5%) [15].

Additionally, RNA ISH has been used to retrospectively investigate clinical response and the extent of RNA expression in comparison to IHC [22]. Although PD-L1 expression was not associated with response to chemotherapy in this study, RNAScope and IHC identified similar numbers of PD-L1 positive patients (33.9% vs 35.1% respectively). A positive relationship was observed between PD-L1 mRNA and PD-L1 protein expression, 67.3% of PD-L1 IHC positive patient samples were PD-L1 RNAScope positive and 88.2% of PD-L1 IHC negative patient samples were also PD-L1 RNAScope negative. These data suggest RNA detection using RNAScope may offer an alternative assay to identify patients suitable for treatment with immune therapy, however further optimisation would be required to define a suitable threshold or cut-off for determining PD-L1 high expression.

Scoring algorithms for PD-L1 status using RNAScope have been previously proposed. For example, greater than 10 punctate dots per cell [23] or greater than 10 dots per cell in greater than 10% of cells have been suggested to be indicative of a cut off between PD-L1 high and PD-L1 low status [15, 24]. These scoring algorithms are rarely derived in a prospective clinical trial setting and rely on retrospective analysis and response data. Thresholds derived in this fashion provide information regarding prognosis or disease classification, they are not however derived from a diagnostic assay linked directly to patient response. To our knowledge there is no data demonstrating a threshold to determine PD-L1 high or PD-L1 low status using the RNAScope assay where the threshold has been derived by maximising the analytical sensitivity and specificity of RNAScope to a commercially available IHC assay.

Here in, PD-L1 RNA expression determined using RNAScope and protein expression determined using a commercially available IHC assay (VENTANA PD-L1 (SP263)) are compared. Data are used to identify a novel threshold to classify tissue as PD-L1 high/low using RNAScope, providing evidence of a potential scoring algorithm that has been derived from a commercially available IHC assay.

## Materials and methods

### Tissue

Ninety (90) commercial clinical cases, thirty (30) for each of NSCLC (BioIVT, West Sussex, UK, ProteoGenex, Inc., Inglewood, CA, USA and Tissue Solutions, Glasgow, UK) HNSCC and UC (Avaden Biosciences, Seattle, WA, USA), previously assessed using the VENTANA PD-L1 (SP263) Assay (Ventana, Tucson, AZ) were chosen to represent a wide range of percentage tumour cell membrane staining (TC_IHC_). This tissue set has previously been assessed using other PD-L1 IHC assays such as Dako PD-L1 IHC 22C3 pharmDx and PD-L1 IHC 28–8 pharmDx assays [12, 25]. Tissue cases included in the analysis represent samples that may be encountered in a routine clinical setting. Tissue was formalin fixed according to industry guidelines [26].

Tissue sections were cut at 4μm thickness. IHC and RNAScope assays were performed on tissue cut within 10 consecutive sections of each other, this minimises the impact of heterogeneity within a tissue block [27]. Tissue sections were assessed by IHC within 3 months of preparation. RNAScope staining was performed within 12 months of tissue sectioning [28].

### RNAScope mRNA *in situ* hybridisation assay

mRNA expression was determined using ISH with the RNAScope 2.5 assay and probe CD274/PD-L1 (Advanced Cell Diagnostics, Hayward, CA) on a Leica Rx instrument (Leica Microsystems Inc., Buffalo Grove, IL) as described previously [21]. Briefly, tissue was pre-treated with heat and protease prior to hybridisation of the target probe. Preamplifier, amplifier and an alkaline phosphatase labelled oligos were sequentially hybridised followed by the application of a chromogenic substrate to produce red punctate dots. Tissue was counter-stained with Mayer’s Haematoxylin.

ACD positive control probe (Peptidylprolyl Isomerase B (Cyclophilin B) (PPIB) and negative control probe (B. subtilis gene dihydrodipicolinate reductase) (dapB) were run on each tissue prior to testing with CD274/PD-L1 probe. Tissue passed quality control if the average number of punctate dots per cell throughout the entire sample were greater than five using the PPIB probe and zero using the dapB probe.

Bright field images were captured using an Aperio AT2 digital slide scanner (Leica Microsystems Inc., Buffalo Grove, IL) and ISH scores were generated using HALO (Indica Labs) image analysis software. Tumour regions of interest were annotated by a board-certified pathologist prior to image analysis in order to standardise tumour areas selected for comparison. Regions of interest totalled 25–50% of the available tumour across the tissue section, the range of cells analysed per tissue section were between 468 to 190,645. Tumour islands were identified and separated from prominent tissue associated stroma. The percentage of tumour cells with any PD-L1 mRNA expression (%TC_mRNA_) and the level of expression (mean punctate dots/tumour cell) using RNAScope were determined.

### Immunohistochemistry

**Fig 1:**
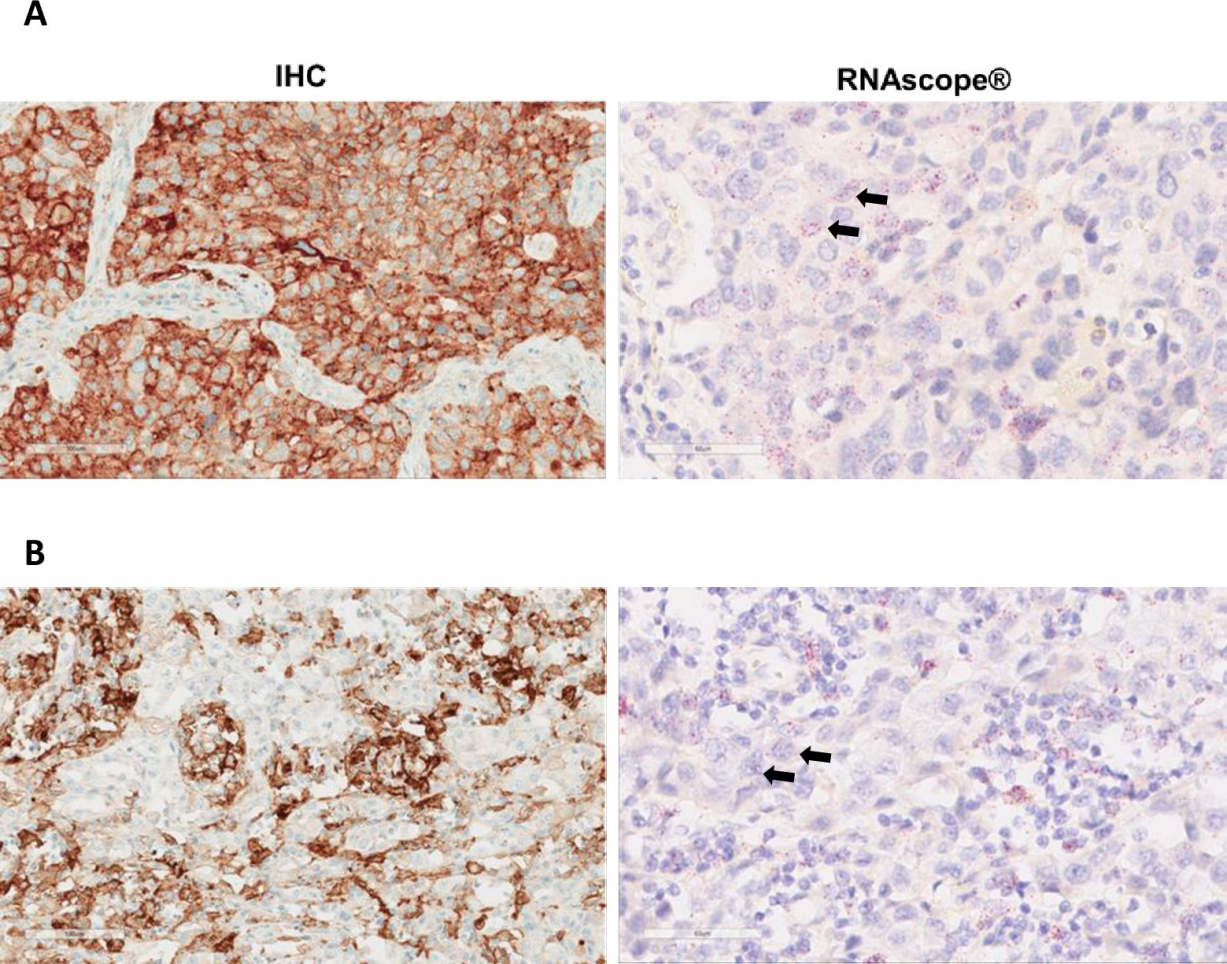
Representative images of 2 NSCLC cases demonstrating varying degrees of %TC_mRNA_ and TC_IHC_ staining. A. Case shows 100% TC _IHC_ staining, 66% TC _mRNA_ staining and 2–3 dots/tumour cell. B. Case shows 70% TC _IHC_ staining, 48% TC _mRNA_ staining and 1–2 dots/tumour cell. Arrows indicate examples of cell nuclei with RNAScope staining.

All samples had previously been stained using the automated VENTANA BenchMark ULTRA platform using the VENTANA PD-L1 (SP263) rabbit mAb assay according to package inserts. Tissue assessment was based on tumour cell membrane staining in all indications. Cases with TC_IHC_ in ≥25% of tumour cells were defined as PD-L1 high, those with TC_IHC_ <25% were defined as PD-L1 low. Membrane staining with a discontinuous, circumferential or basolateral pattern was included in analysis. Tumour infiltrating lymphocyte (TIL) staining was not assessed. Samples were read by a single pathologist trained by the manufacturer in a Clinical Laboratory Improvement Amendments (CLIA) program–certified laboratory (Hematogenix Laboratory Services, IL, US). Equal numbers of PD-L1 high and PD-L1 low cases from each indication were selected for RNAScope staining based on the 25% PD-L1 TC_IHC_ cut off. Representative images of IHC and RNAScope staining are shown in Fig 1.

### Statistics

Differences in %TC_mRNA_ and dots/tumour cell between PD-L1 high (TC_IHC_ ≥25%) and PD-L1 low (TC_IHC_ <25%) groups were assessed using t-tests (Microsoft Excel 2016).

For each indication, strip charts and a receiver-operating characteristic (ROC) analysis (R version 3.5.0, package pROC [29]) were prepared to identify thresholds for patient classification using %TC_mRNA_ and dots/tumour cell, with reference to TC_IHC_ ≥25%.

## Results

### Tissue staining quality

Ninety (90) FFPE cases from NSCLC, HNSCC and UC were assessed for analysis with RNAScope. Eighty-six of the tumours showed PD-L1 staining with RNAScope. One UC case was not analysed due to the absence of tumour. Two NSCLC cases and one HNSCC case were not analysed due to insufficient/absent staining using RNAScope positive control probes (QC fails).

Twenty-eight NSCLC cases were successfully stained using both SP263 and RNAScope assays. Twenty-nine HNSCC cases were successfully stained using both SP263 and RNAScope assays. Twenty-nine UC cases were successfully stained using both SP263 and RNAScope assays.

### Comparison of mRNA ISH and IHC staining

**Fig 2:**
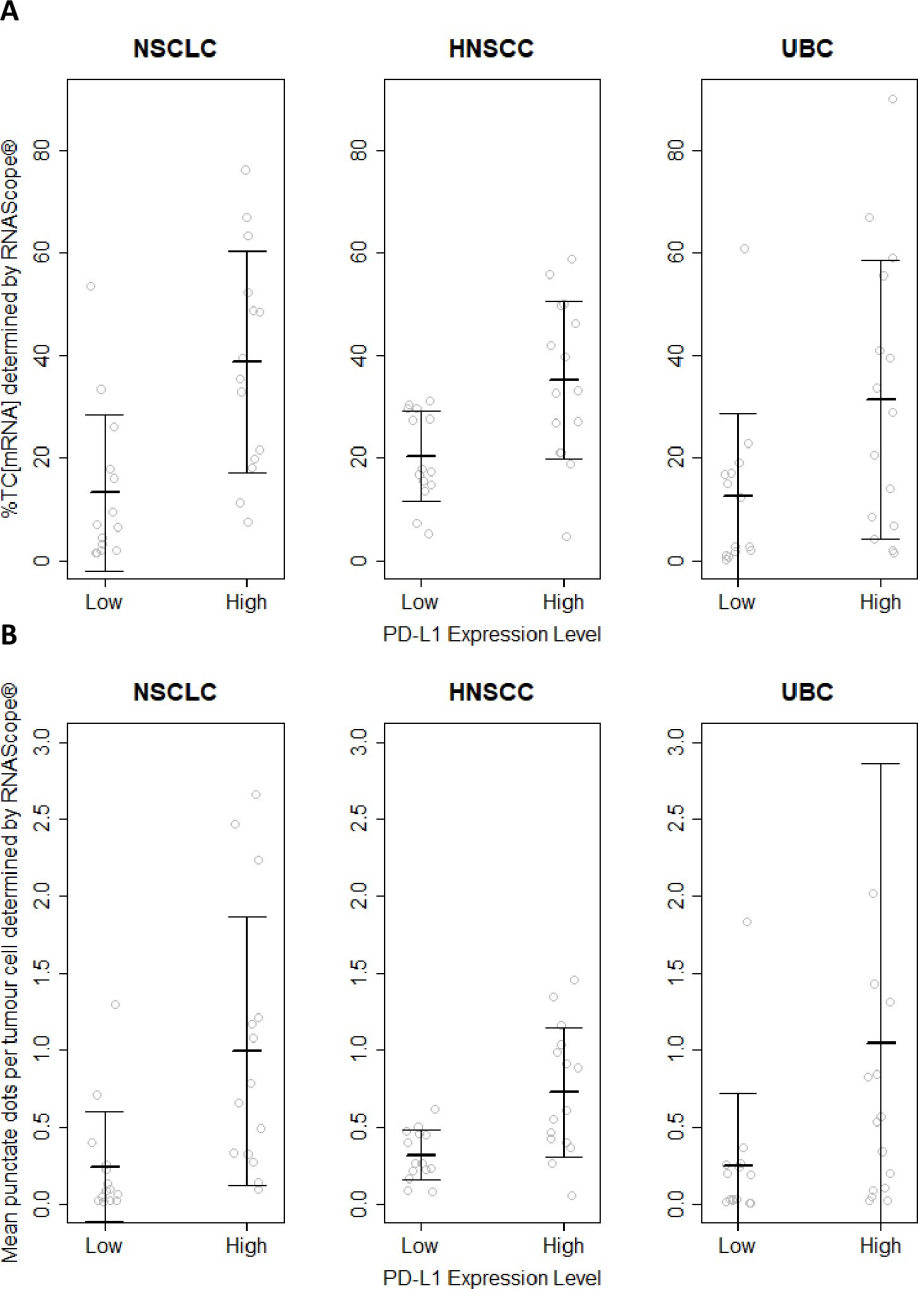
**%TC**_**mRNA**_ **(A) and dots /cell (B) comparison to TC**_**IHC**_ **<25% (Low) vs ≥25% (High) PD-L1 expression in NSCLC, HNSCC and UC.** %TC_mRNA_ and the number of punctate dots per cell were grouped according to PD-L1 expression determined by IHC. %TC_mRNA_ demonstrated statistical significance (at α = 0.05) in the PD-L1 high vs PD-L1 low groups for NSCLC, HNSCC and UC. The number of punctate dots/tumour cell was statistically significantly higher in the PD-L1 high vs PD-L1 low groups for NSCLC and HNSCC but not UC. Fig 2B UC plot, PD-L1 high, one outlier (7.24 dots/tumour cell) is not shown.

The %TC_mRNA_ staining for PD-L1 mRNA ranged from 1.31% to 75.99% in NSCLC, from 4.55% to 58.63% in HNSCC and 0.19% to 89.8% in UC (Fig 2). The dots per cells ranged from 0.02 to 2.66 in NSCLC, from 0.08 to 1.45 in SCCHN and from 0.0021 to 7.25 in UC (Fig 2).

%TC_mRNA_ was significantly higher (at α = 0.05) in the TC_IHC_≥25% vs <25% groups for NSCLC (38.7 vs 13.1% p = 0.0014), HNSCC (35.1 vs 20.2 p = 0.0040) and UC (31.4 vs 12.4% p = 0.030).

Dots/tumour cell was significantly higher in the TC_IHC_≥25% vs <25% groups for NSCLC (0.99 vs 0.24 p = 0.0083) and HNSCC (0.73 vs 0.32 p = 0.0023) but not UC (0.25 vs 1.0 p = 0.12). Data shown in Table 1 and Fig 2.

**Table 1:** Mean %TC_mRNA_ and dots/tumour cell staining in PD-L1 IHC high and low groups.

	Mean %TC_mRNA_ staining		Mean dots/tumour cell staining	
Indication	PD-L1 IHC low	PD-L1 IHC high	PD-L1 IHC low	PD-L1 IHC high
NSCLC	13.1	38.7	0.24	0.99
HNSCC	20.2	35.1	0.32	0.73
UC	12.4	31.4	0.25	1.00

For each indication mean % TC mRNA staining and dots/tumour cell staining are shown for PD-L1 IHC high and PD-L1 IHC low groups.

### mRNA threshold and PD-L1 status

**Fig 3:**
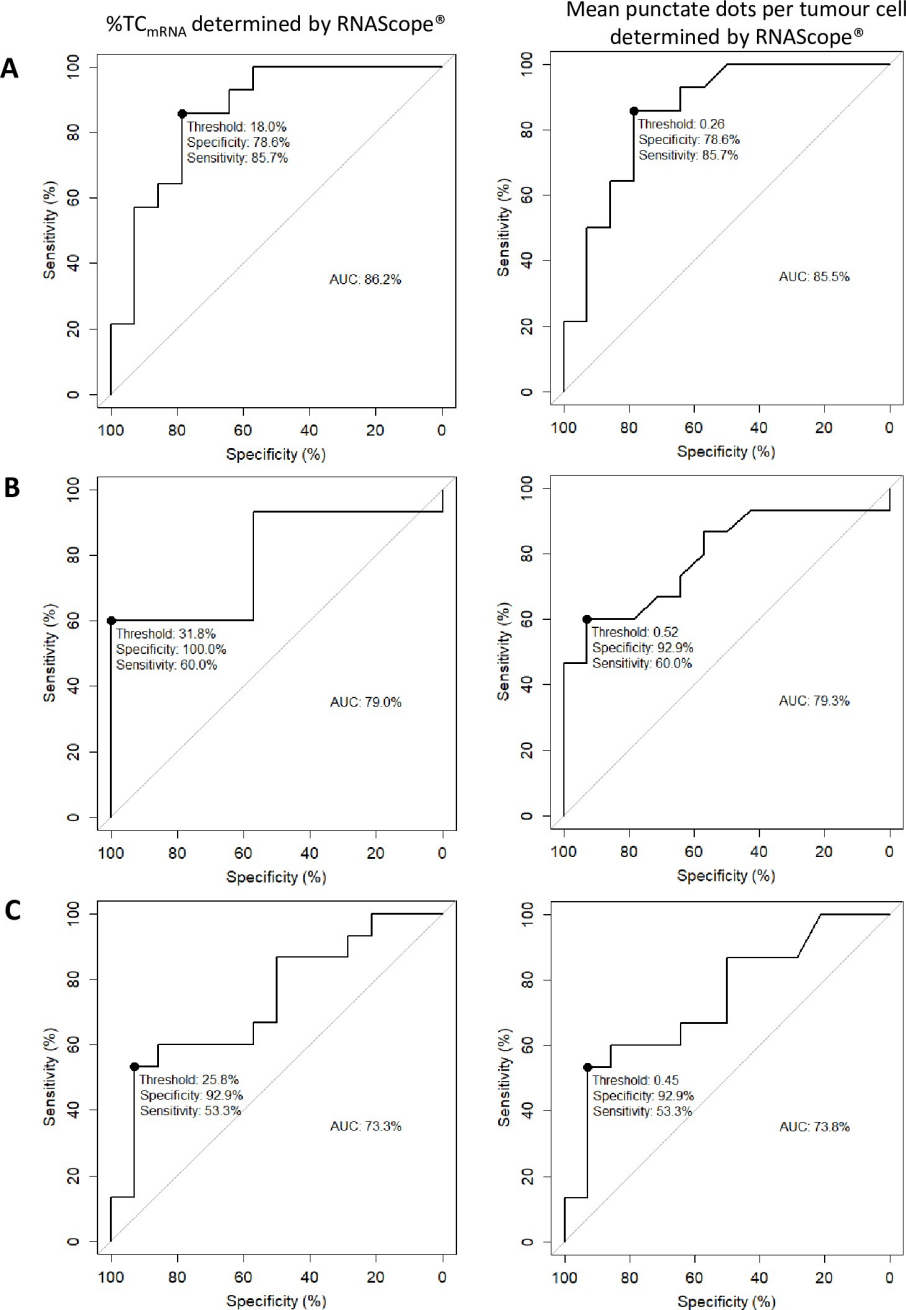
**ROC analysis was performed using %TC**_**mRNA**_ **and dots/tumour cell in NSCLC (A), HNSCC (B) and UC (C).** Based on the highest sensitivity and specificity against IHC (SP263) using a 25% cut off, ROC analysis was used to identify PD-L1High/Low thresholds for RNAScope. Maximum sensitivity and specificity are identified on each plot (•) and the threshold for %TC_mRNA_ and dots/tumour cell given. The area under each curve (AUC) is calculated and for all indications demonstrates fair accuracy of RNAScope compared to IHC.

A ROC analysis was used to determine an analytical cut off for %TC_mRNA_ and average dots per cell using RNAScope. Analytical cut off determination was based on the highest sensitivity and specificity of the RNAScope assay against IHC (SP263) using a 25% tumour cell staining cut off. For %TC_mRNA_, the ROC analysis identified thresholds of: NSCLC 18.0%, HNSCC 31.8%, UC 25.8%. For dots/tumour cell, ROC analysis identified thresholds of: NSCLC 0.26, HNSCC 0.52, UC 0.45 (Fig 3).

## Discussion

PD-L1 mRNA was detected across tissue (the percentage of tumour cells with any RNA staining) and expression level (the amount of staining per tumour cell) demonstrating positive association with PD-L1 status as determined by IHC. The %TC_mRNA_ and dots/tumour cell in NSCLC, SCCHN and UC were higher in tissue sections showing high PD-L1 protein expression.

Tissue for this study represented a variety of indications and sample acquisition methods which routine laboratories may encounter. mRNA was quantifiable in 96% of cases, demonstrating, as has been shown previously, that routine tissue processing is suitable for the preservation of RNA [18]. mRNA was not quantifiable in 4% of cases due to the absence of tumour in one case and the absence of positive control probe staining in 3 cases. The automated RNAScope assay, used to generate this data is similar to other ISH assays and requires overnight processing; this is longer in comparison to IHC. Both assays can however fit within a standard routine pathology laboratory shift pattern. RNAScope is automated on instruments widely used in routine laboratories and analysis can be performed by standard microscopy, potentially facilitating deployment within a clinical pathology department. Further optimisation of ISH may reduce turnaround time further, making the assay more appealing for routine use.

The VENTANA PD-L1 (SP263) rabbit mAb assay was used in this study. IHC assays such as Dako PD-L1 IHC 22C3 and 28–8 assays have been shown to demonstrate good concordance with each other [11, 12, 25]; the sensitivity and specificity of RNAScope could therefore be inferred to extended beyond the VENTANA PD-L1 (SP263) rabbit mAb assay to the Dako PD-L1 IHC 22C3 and 28–8 assays. The close association of RNA expression detection to protein expression determined by IHC indicates RNAScope may be suitable for use in routine clinical diagnosis and could be a viable alternative assay to IHC following further analytical and clinical validation.

Threshold optimisation for %TC_mRNA_ and mean dots/tumour cell, results in high specificity to IHC PD-L1 classification, but only moderate sensitivity. The RNAScope assay, if compared to VENTANA PD-L1 (SP263) rabbit mAb assay (as a gold standard), would select a marginally different PD-L1 high patient population using the cut offs derived here. For NSCLC, %TC_mRNA_ threshold at 18% and dots/tumour cell cut off at 0.26, 85.7% of PD-L1 high IHC cases would also be PD-L1 high by RNAScope and 78.6% of PD-L1 low IHC cases would also be classified as PD-L1 low by RNAScope. For HNSCC, using %TC_mRNA_ cut off at 31.8% or 0.5 dots per cell, 60% of PD-L1 high IHC cases would also be PD-L1 high by RNAScope. Using these cut off values RNAScope showed 100% or 93% specificity for %TC_mRNA_ and dots per cell respectively. For UC using %TC_mRNA_ cut off 25.8% or 0.45 dots/tumour cell, 53.3% of PD-L1 high IHC cases would also be PD-L1 high by RNAScope and 92.9% of PD-L1 low IHC cases would also be classified as PD-L1 low by RNAScope. The moderate sensitivity of RNAScope compared with IHC results in selection of a larger PD-L1 low patient population, the clinical response of which is unknown. No data regarding patient response was available in this study, and further clinical validation of mRNA detection using RNAScope to select patients is required.

These data were derived using the anti-human PD-L1 rabbit mAb (SP263), PD-L1 high staining was defined as ≥25% tumour cell staining at any intensity. Alternative thresholds in different indications may yield different results [30]. Evidence shows that immune cell infiltrate and PD-L1 staining of these cells is an important part of determining patient response [31], but accurate assessment of this cell type may be subject to inter reader variability [32]. Of note, in UC, immune cell staining by IHC or RNAScope was not assessed. Immune cells could not be accurately identified by image analysis and therefore PD-L1 mRNA expression could not be quantified in these cells. The challenge associated with immune cell identification and scoring could be overcome using RNAScope and we propose a number of strategies here.

RNAScope could be used to aid the identification of immune cells by staining specific immune cell markers, such as CD4 and CD8. Combining RNAScope with IHC could potentially enable better patient stratification and therefore efficacy to treatments which target immune checkpoint blockade. RNAScope has been developed previously as a dual stain utilising detection of RNA and protein [33]. Challenges associated with manual analysis and assessment of immune cells may however remain, due to the diffuse distribution of immune cells across a tissue section. Further analysis of mRNA expression (using RNAScope) in immune infiltrate compared to IHC may provide further confirmation of IHC vs RNAScope concordance.

Alternatively, a multiplex assay targeting PD-L1 transcripts and protein by RNAScope and IHC could be used to identify subsets of patients, for example those that are PD-L1 IHC low, but mRNA expression high, may respond differently to PD-L1 targeted therapies. Additionally, a multiplex approach will reduce the tissue requirements of a diagnostic assay on small, precious clinical samples [34].

Here, RNA expression was measured using a combination of a commercially available assay and image analysis software. Quantifying the amount of RNA across a whole tumour section in individual tumour cells is however challenging. Manually quantifying mRNA expression requires an extensive amount of time and may be prone to error due to the complexity associated with the number of tumour cells present in a tissue section and the relationship with the surrounding stroma. One approach to simplify analysis is to enumerate a small number of representative cells, this approach may not be ideal, potentially excluding a large amount of useful data. Alternatively, as employed in this study, RNAScope may be exploited in combination with image analysis software, allowing large numbers of tumour and immune cells to be analysed. Image analysis may prove particularly useful to standardise the analysis of immune cells, especially if a multiplex assay can be employed as described above. Image analysis will also enable any heterogeneity within the expression profile to be investigated and analysed. Accurate determination of PD-L1 staining in large numbers of tumour cells, identification of immune cells and identification of heterogeneity may be useful to direct patient treatment options especially in cases which are equivocal or borderline [21].

Data presented here was quantified using commercially available image analysis software. Analysis is based on predefined rules and is driven by a fixed algorithm. The use of machine learning to aid clinical diagnosis has been explored using immunohistochemistry in breast cancer [35]. RNAScope and machine learning may be ideally suited to one another for whole tissue section analysis in that the ISH assay produces a high volume, quantifiable output. Machine learning could be adopted in a clinical setting to elucidate temporal and spatial gene expression. Implementing machine learning to make treatment decisions would however require significant validation.

## Conclusions

We have shown detection of PD-L1 RNA in commercially available clinical samples using RNAScope demonstrates high specificity and moderate sensitivity with protein detection using a commercially available PD-L1 IHC assay (Ventana SP263). Both assays would select marginally different patient populations. Due to the moderate sensitivity of RNAScope compared to IHC, RNAScope identifies a population of cases across NSCLC, HNSCC and UC as PD-L1 high that would not be PD-L1 high by IHC. The response of these patients to treatment with PD-L1 immunotherapy is unknown. The thresholds presented here for RNAScope are for the first time derived using a commercially available IHC assay as a reference but further validation with patient outcome data is required.
